# The efficacy and safety of Xueshuantong (lyophilized) for injection in the treatment of unstable angina pectoris: A systematic review and meta-analysis

**DOI:** 10.3389/fphar.2023.1074400

**Published:** 2023-04-07

**Authors:** Junyu Xi, Ruili Wei, Xin Cui, Yi Liu, Yanming Xie

**Affiliations:** Institute of Basic Research in Clinical Medicine, China Academy of Chinese Medical Sciences, Beijing, China

**Keywords:** Xueshuantong for injection, unstable angina pectoris, meta-analysis, trial sequential analysis, GRADE

## Abstract

**Objective:** Xueshuantong (lyophilized) for injection (XST) is an effective botanical drug for treating unstable angina pectoris (UAP). However, a meta-analysis of XST combined with conventional treatment (CT) against UAP has not been conducted. Therefore, this study aimed to investigate the effectiveness and safety of XST combined with CT for UAP patients compared to CT alone.

**Methods:** Randomized controlled trials (RCT) of XST in UAP patients were retrieved from the Cochrane Library, PubMed, Web of Science, EMBASE, CNKI, VIP, Wanfang, and Chinese Biological Medicine Database databases. A meta-analysis was performed using Revman 5.4 and Stata 16.0, and the quality of the included literature was evaluated based on the Cochrane risk-of-bias 2.0 (RoB2.0) tool. The aggregate 95% confidence intervals (CIs), mean difference (MD), and relative risk (RR) estimates were calculated. A GRADE assessment was performed using GRADEprofiler 3.6, and trial sequent analysis was performed using TSA 0.9.

**Results:** Thirty-four studies involving 3,518 patients were included in the analysis. The combination of CT with XST improved the comprehensive clinical efficacy (RR = 1.22, 95% CI: 1.18–1.26, *p* < 0.00001) and ECG improvement (RR = 1.24, 95% CI: 1.18–1.31, *p* < 0.00001). The frequency of angina attacks was lower (MD = −0.73, 95% CI: −0.92 to −0.55, *p* < 0.00001), and the duration was shorter (MD = −1.08, 95% CI: −1.44 to −0.72, *p* < 0.00001) in the group that received CT combined with XST compared to the one without XST. Total cholesterol levels (MD = −1.30, 95% CI: −1.83 to −0.78, *p* < 0.00001) and triglyceride levels (MD = −0.76, 95% CI: −0.93 to −0.59, *p* < 0.00001) were lower in patients who received CT in combination with XST than those who received CT alone. CT combined with XST reduced whole blood viscosity (MD = −0.72, 95% CI = −0.99 to −0.44, *p* < 0.00001) and plasma viscosity (MD = −0.24, 95% CI: −0.46 to −0.03, *p* = 0.03). There was no statistically significant difference in the incidence of cardiovascular events or adverse events among patients treated with the combination of XST and CT compared to CT alone. The GRADE assessment indicated that the composite quality of the evidence was low. The trial sequent analysis showed an adequate sample size and stable findings for the clinical efficacy of CT combined with XST for unstable angina.

**Conclusion:** The present systematic review and meta-analysis conditionally indicate that XST combined with CT improved the clinical outcomes of patients with unstable angina more than CT alone with a better safety profile. However, the results need further validation due to limitations in the quality of the included studies.

**Systematic Review Registration:** https://www.crd.york.ac.uk/PROSPERO/, identifier CRD42022357395.

## 1 Introduction

Unstable angina pectoris (UAP) is integral to acute coronary syndrome (ACS). An epidemiological investigation in China reported that 122 of 100,000 people had coronary heart disease, imposing a huge burden on families and society (Summary of Chinese Cardiovascular Health and Disease report 2019, 2020). As a non-ST segment-elevated acute coronary syndrome, UAP can develop into acute myocardial infarction and is the leading cause of death in patients with coronary heart disease. The main UAP treatments in modern medicine include anti-myocardial ischemia, antiplatelet, anticoagulation and statin medications, and revascularization (Guidelines for diagnosis and treatment of non-ST segment elevation acute coronary syndrome 2016, 2017). However, the long-term use of these drugs produces side effects and drug resistance, affecting treatment efficacy. Chinese medicine has been used in clinical practice for over 3,000 years (Jia et al., 2017; Zhang et al., 2017). Chinese patent medicine (CPM) refers to drugs prepared from various botanical drugs through unique methods and specific combinations. Modern evidence-based evaluation methods and basic research evidence have confirmed that CPM is advantageous and safe for treating UAP and is widely used for treating UAP. Chinese botanical drug injections are fast-acting and have better bioavailability than general CPM (Li et al., 2022).

Xueshuantong (lyophilized) for injection (XST) is a standardized botanical drug in the *Pharmacopoeia of the People’s Republic of China*. It is listed in the 2018 National List of Essential Medicines as one of the most popular Chinese botanical drug injections for activating blood circulation and resolving blood stasis. *Panax notoginseng* saponins were isolated from the root and rhizome of *Panax notoginseng* (Burk.) F. H. Chen [Araliaceae; Notoginseng Radix], is the primary active component of XST (Shen et al., 2017). *Panax notoginseng* is a well-known botanical drug. It has the ability to invigorate blood, remove blood stasis, reduce swelling and relieve pain. Pharmaceutical manufacturer obtained 250 g of total saponins of *panax notoginseng*, and an appropriate amount of water was added for injection to dissolve it, followed by activated carbon. After homogenizing and filtering, water was injected into the specified volume. Then, 20% sodium citrate solution was added to adjust the pH value to 5.5–7.0, followed by filtering, filling, and freeze drying, to obtain XST. There are five main components of PNS: ginsenoside R1, ginsenoside Rg1, ginsenoside Rd, ginsenoside Re, ginsenoside Rb1. Previous studies confirmed that *Panax ginseng* total saponin could correct intracellular calcium ion (Ca2^+^) overload in cardiomyocytes by increasing the calcium pump activity on the sarcoplasmic reticulum (SR) membrane of cardiomyocytes, thereby increasing left ventricular myocardial energy (Feng and Jiang, 1998). Animal experiments established that *Panax ginseng* total saponin could inhibit nuclear factor (NF)-κB activation in neutrophils and reduce intercellular adhesion molecule (ICAM)-1 expression and neutrophil infiltration. Thus, it improves myocardial microcirculation, narrowing the infarct range of myocardial ischemia and effectively counteracting T-wave elevations due to acute myocardial ischemia (Tang et al., 2002; Li and Wang, 2016). The literature search yielded a previous meta-analysis of XST in treating UAP (Gao et al., 2019), but with certain limitations. The study did not consider long-term outcomes, such as the incidence of adverse cardiovascular events. The level of evidence was not evaluated, and the findings were not updated with the results of clinical trials conducted in recent years. Simultaneously, various dosage forms of XST were analyzed without any quality evaluation or sample size estimation. The current analysis used only studies of high-purity XST freeze-dried powder for the test sequential analysis to explore the stability of the study conclusions. GRADE was also used to evaluate the evidence quality, hoping to provide physicians with an accurate and objective reference on XST.

## 2 Methods

This meta-analysis was conducted and reported according to the PRISMA 2020 statement (Page et al., 2021) (Supplementary Material S1). The study protocol was registered with PROSPERO (registration number: CRD42022357395).

### 2.1 Eligibility criteria

#### 2.1.1 Type of study

Randomized controlled trials (RCTs) were included, and the subjects met the diagnostic criteria of unstable angina pectoris (Chinese Medical Association, 2007).

#### 2.1.2 Type of intervention

The test group was treated with XST, or XST was added to the control group. The control group was treated with routine medications (vasodilators, anticoagulants, antihypertensive drugs, and statins) (Chinese Medical Association, 2007) or other drugs.

#### 2.1.3 Types of outcomes

The main index used to evaluate the effectiveness of this study was the comprehensive clinical efficacy, which referred to the sum of the percentage of patients whose curative effects were “significantly effective” and “effective” (FDA, 2007). Among them, a significant effect indicated that the number of angina pectoris events was reduced by more than 80%, and more than two grades improved the UAP grade. “Effective” suggested that the number of angina pectoris events decreased by 50% to more than 80%, and the UAP grade was improved by more than 1. The secondary outcomes were ECG improvement, the number of angina attacks, angina duration, blood lipid levels, and blood rheology assessments. ECG improvement was either the return of the ECG to normal, ST segment recovery >0.05 mV or the shallowing amplitude of the inverted T wave of the main lead is greater than 25% of the original or a change in the T wave from flat to upright. The primary outcome index used to evaluate safety was the incidence of adverse reactions, and the secondary outcome index was the incidence of adverse cardiovascular events.

### 2.2 Retrieval strategy

We searched PubMed, Embase, Web of Science, Cochrane Library, China National Knowledge Infrastructure (CNKI), VIP Chinese Sci-tech Periodical Database (VIP), Chinese Biological Medicine Database (CBM), and the Chinese Wan Fang Database (Wan fang) from the inception of the database to October 2022. The retrieval strategies for each database are depicted in Supplementary Material S2.

### 2.3 Selection of research studies

The study selection process followed the PRISMA statement guidelines (Page et al., 2021). JX and XC independently screened the literature with predefined study selection criteria. The literature retrieved by subject terms was first imported into NoteExpress software for weight-checking, followed by a preliminary screening based on the titles of the publications. Afterward, the abstracts were read to exclude literature that did not meet the inclusion criteria. Finally, the full text of the studies was used to finalize inclusion in the current study. Disagreements were resolved by discussing with a third party. The data extraction table was created using Excel. The extracted data mainly included the first author, year of publication, the baseline characteristics of the study population, interventions (including dose and duration of treatment), and outcome indicators.

### 2.4 Methodological quality evaluation

Two investigators independently evaluated the methodological quality of the studies from relevant specialties, and a third party was consulted to resolve any disagreement. The quality of the included literature was assessed using the Cochrane risk-of-bias 2.0 (RoB2.0) tool (Liu et al., 2019). It focuses on randomization process, deviations from intended interventions, missing outcome data, measurement of the outcome, and selection of the reported result. Five aspects were evaluated, and the included studies were judged as having a low or high risk of bias, or some concerns.

### 2.5 Statistical analysis

Data analysis was performed using Revman 5.3 statistical software provided by the International Cochrane Collaboration. Mean difference (MD) (continuous variable) and relative risk (RR) (dichotomous variable) were selected as effect sizes, and 95% confidence intervals (CI) were utilized to express interval estimates. When the heterogeneity among the studies was small (*p* ≥ 0.1, *I* ^2^ ≤ 50%), the fixed-effects model was selected for analysis. The reason was analyzed when the heterogeneity of each study was significant. If it was caused by clinical factors or research methods, subgroup or sensitivity analysis was performed. If the heterogeneity was still significant after analysis (*p* < 0.1, *I* ^2^ > 50%), the random-effects model was selected for analysis. When more than 10 articles were included in the outcome index, a funnel chart was made using Stata.16.0 to analyze whether the studies had publication bias. TSA0.9 was used to sequentially analyze comprehensive clinical efficacy to evaluate whether the sample size of this meta-analysis was sufficient and whether there were false-positive results.

### 2.6 Assessment of evidence quality

JLX and RLW independently assessed the evidence quality according to previous rating standards (Balshem et al., 2011; Guyatt et al., 2011). The quality of the evidence included in the results was classified as high, medium, low, or very low. RCTs were initially classified as having high-quality evidence. The quality of each outcome was further graded according to five factors: deviation risk, inconsistency, indirectness, inaccuracy, and publication deviation. GRADEpro 3.6.1 software was utilized for data analysis and synthesis.

## 3 Results

### 3.1 Literature screening process

**FIGURE 1:**
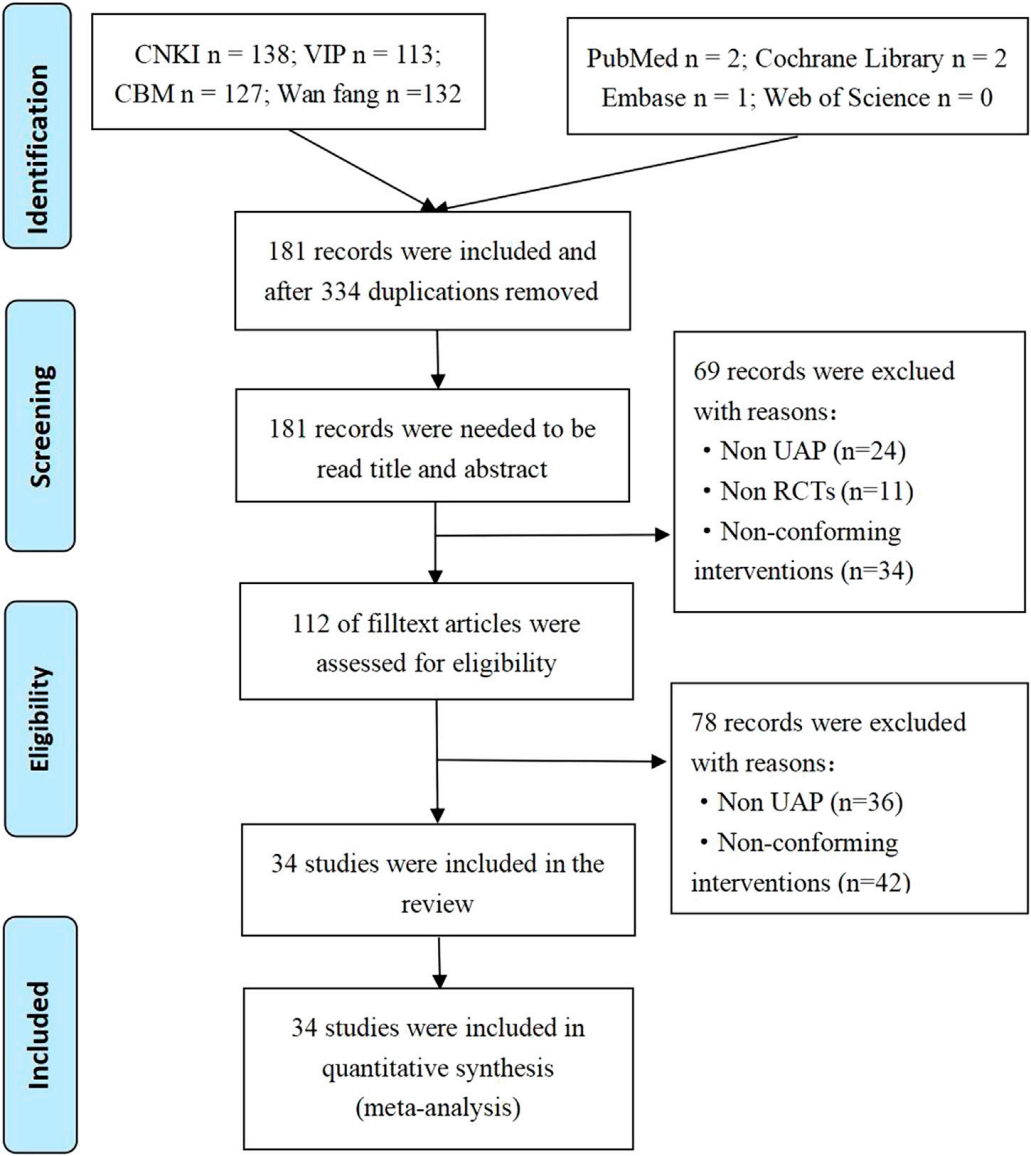
Flowchart of article selection process.

We systematically searched the databases and obtained 515 potentially related original studies. Of them, 334 duplicate studies were deleted, and the titles and abstracts of the remaining 181 articles were examined. Sixty-nine studies were deleted since they did not meet the eligibility criteria. Of these, 11 did not meet the inclusion criteria for the type of research, 34 did not meet the intervention criteria, and 24 were non-UAP studies. After further screening the full text of the studies, 78 articles were excluded since the study or intervention measures did not meet the inclusion criteria. Finally, a total of 34 original studies were included in the meta-analysis. The PRISMA flow chart study selection is represented in Figure 1.

### 3.2 Research and participant characteristics

The 34 included studies were all Chinese literature (Luan and Sun, 2005; Zeng, 2007; Xie and Yan, 2008; Du, 2009; Liang, 2010; Pan, 2010; Wang, 2010; Liang and Wu, 2011; Ou, 2011; Qi and Dong, 2011; Sun et al., 2011; Zhang, 2011; Zhe, 2011; Zheng, 2011; Chen et al., 2012; Ju and Dong, 2012; Wang et al., 2012; Ming, 2013; Pan et al., 2013; Li, 2014; Luo, 2014; Tan, 2014; Wang et al., 2014; Yan and Yu, 2014; Chen, 2015; Dong and Yu, 2015; Guan et al., 2015; Fei et al., 2016; Feldavus and Sarchen, 2016; Wang, 2016; Zhong et al., 2016; Huang, 2017; Wang and Wei, 2018; Zang, 2020). The total sample size included 3,518 subjects, 1,765 in the experimental group and 1,753 in the control group. The maximum sample size of the trial group was 200, and the minimum sample size was 15. In the control group, the maximum sample size was 200, and the minimum sample size was 15. The shortest intervention period was 7 days while the longest was 21 and 14 days were the main ones (11/42, 52.94.19%). The mean age of the participants ranged from 45 to 79.1 years, as shown in Table 1.

**TABLE 1:** Data analysis of included studies.

Study	Sample size			Gender (T/C)	Age/(year) (T/C)	Interventions		Duration/day	Outcomes
	T	C	Total			C	T		
Luan and Sun. (2005)	30	30	60	T: 19/11	63	CTs	CTs + XST 500 mg	15	①②
				C: 16/14					
Zeng (2007)	40	40	80	T: 28/12	T: 58.6	CTs	CTs + XST 500 mg	15	①⑤
				C: 27/13	C: 59.1				
Xie and Yan. (2008)	46	45	91	T: 46/36	T: 54.2 ± 9.0	CTs	CTs + XST 450 mg	7	①②⑤⑦
				C: 45/35	C: 54.1 ± 9.2				
Du (2009)	29	31	60	T: 15/15	T: 62	CTs	CTs + XST 400 mg	10	①②⑦⑧
				C: 16/14	C: 60				
Liang (2010)	60	60	120	66/54	54	CTs	CTs + XST 500 mg	21	① ②
Pan (2010)	98	98	196	T: 51/47	T: 62.3 ± 7.6	CTs	CTs + XST 500 mg	12	①
				C: 52/46	C: 61.4 ± 8.1				
Wang (2010)	58	56	114	T: 37/21	T: 63.4 ± 8.7	CTs	CTs + XST 500 mg	14	①
				C: 30/26	C: 64.1 ± 9.2				
Liang and Wu. (2011)	30	30	60	T: 18/12	T: 57.8 ± 10.4	CTs	CTs + XST 500 mg	10	①
				C: 16/14	C: 58.1 ± 10.9				
Ou (2011)	45	45	90	T: 29/16	T: 57.5 ± 6.1	CTs	CTs + XST 500 mg	14	①②
				C: 32/13	C: 56.3 ± 55.6				
Qi and Dong. (2011)	200	200	400	T: 110/90	T: 67.40 ± 10.21	CTs	CTs + XST 400 mg	14	①②
				C: 112/88	C: 66.93 ± 10.21				
Sun et al., 2011	48	48	96	55/41	54	CTs	CTs + XST 400 mg	14	①⑦
Zhang 2011	36	35	71	46/25	64	CTs	CTs + XST 450 mg	14	①②
Zhe (2011)	15	15	30	T: 15/10	UK	CTs	CTs + XST 500 mg	15	①②
				C: 15/10					
Zheng (2011)	40	40	80	T: 17/23	66.98 ± 12.12	CTs	CTs + XST 120 mg	14	①
				C: 19/21					
Chen et al. (2012)	40	40	80	T: 18/22	T: 54	CTs	CTs + XST 500 mg	14	①②⑥
				C: 17/23	C: 56				
Ju and Dong. (2012)	55	55	110	72/38	58	CTs	CTs + XST 500 mg	12	①
Wang et al. (2012)	30	30	60	UK	UK	CTs	CTs + XST 500 mg	14	①②④③
Ming (2013)	47	46	93	T: 28/19	T: 62.8 ± 5.9	CTs	CTs + XST 500 mg	21	①
				C: 28/18	C: 62.1 ± 3.9				
Pan et al. (2013)	30	30	60	UK	UK	CTs	CTs + XST 350 mg	—	①⑦
Li (2014)	30	30	60	T: 20/10	T: 62.5 ± 4.7	CTs	CTs + XST 400 mg	14	①②③ ④⑦⑧
				C: 21/9	C: 68.1 ± 4.8				
Luo (2014)	75	75	150	T: 40/35	T: 60.5 ± 10.7	CTs	CTs + XST 500 mg	14	①③⑦
				C: 39/36	C: 61.3 ± 10.5				
Tan (2014)	30	30	60	39/21	55.4 ± 7.5	CTs	CTs + XST 500 mg	14	①②
Wang et al. (2014)	52	52	104	T: 25/25	T: 57.6 ± 4.6	CTs	CTs + XST 500 mg	14	①②
				C: 26/24	C: 55.9 ± 5.4				
Yan and Yu. (2014)	45	45	90	T: 25/20	T: 55.9 ± 6.3	CTs	CTs + XST 500 mg	14	①②⑦
				C: 26/19	C: 56.0 ± 6.1				
Chen (2015)	26	26	52	UK	61.2 ± 4.3	CTs	CTs + XST 300 mg	10	①
Dong and Yu. (2015)	60	60	120	T: 31/29	T: 62.9 ± 4.1	CTs	CTs + XST 450 mg	14	⑤⑥
				C: 36/24	C: 65.7 ± 4.5				
Guan et al. (2015)	54	54	108	T: 39/15	T: 53.26 ± 3.49	CTs	CTs + XST 400 mg	15	①⑤⑥
				C: 45/9	C: 56.91 ± 4.60				
Feldavus (2016)	130	130	260	T: 76/54	T: 53.7	CTs	CTs + XST	7	①⑥
				C: 69/61	C: 52.5				
Fei et al. (2016)	72	72	144	T: 52/20	T: 61 ± 3	CTs	CTs + XST 400 mg	15	①⑥
				C: 47/25	C: 60.6 ± 2.9				
Wang et al. (2014)	38	29	67	42/35	60.0 ± 5.2	CTs	CTs + XST 500 mg	12	①②⑦
Zhong et al. (2016)	25	25	50	28/22	45–70	CTs	CTs + XST 250 mg	14	①②
Huang (2017)	48	48	96	53/43	65.3 ± 5.9	CTs	CTs + XST 450 mg	14	①
Wang and Wei. (2018)	60	60	120	T: 29/31	T: 58.3 ± 7.1	CTs	CTs + XST 500 mg	14	①②
				C: 32/28	C: 57.2 ± 7.3				
Zang (2020)	43	43	86	T: 28/15	T: 54.65 ± 3.24	CTs	CTs + XST 250 mg	14	①②③
				C: 25/18	C: 57.65 ± 6.23				④⑥⑦

T, treatment group; C, control group; XST, Xueshuantong injection; CTs, Conventional treatments: vasodilators, anticoagulants, antihypertensive drugs, statins; UK, unknown; ①: Overall response rate; ②: Effectiveness in ECG; ③: Duration of angina pectoris; ④: Frequency of angina pectoris; ⑤: Hemorheology level; ⑥: Blood lipid level; ⑦: Adverse events; ⑧: Adverse cardiovascular events.

### 3.3 Methodological quality

**FIGURE 2:**
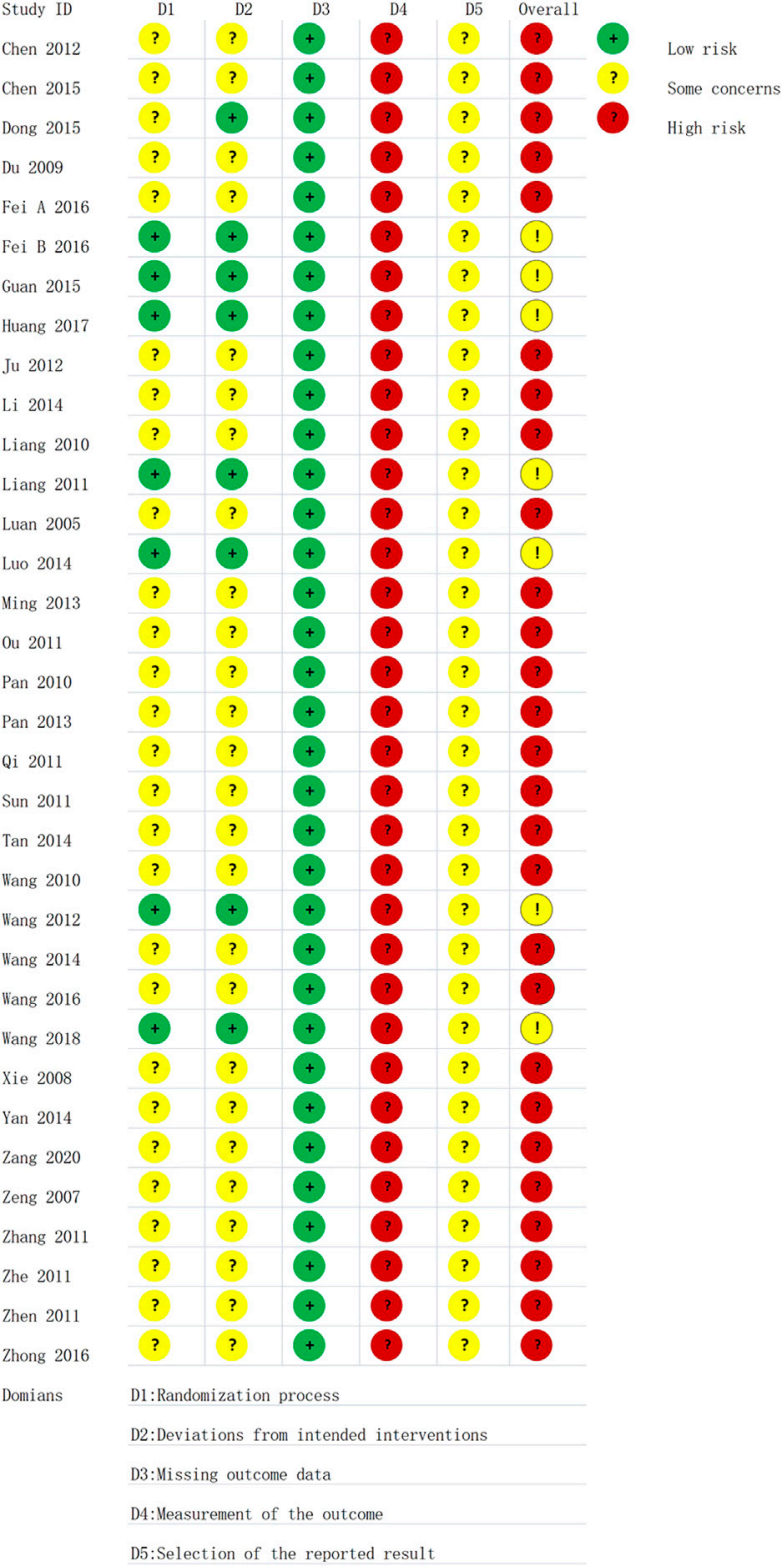
Risk of bias in each trial according to the Cochrane Ro B2 tool.

All included studies were RCTs, seven of which reported specific randomization methods, particularly the random number table method. The others only mentioned randomization. One study said that patients provided written informed consent. Sample size estimates and protocol concealment for all studies were not adequately reported. The presence of other biases was unclear in all of the studies. The risk of bias assessment is shown in Figure 2.

### 3.4 Meta-analysis

#### 3.4.1 Primary efficacy outcome

##### 3.4.1.1 Comprehensive clinical efficacy

**FIGURE 3:**
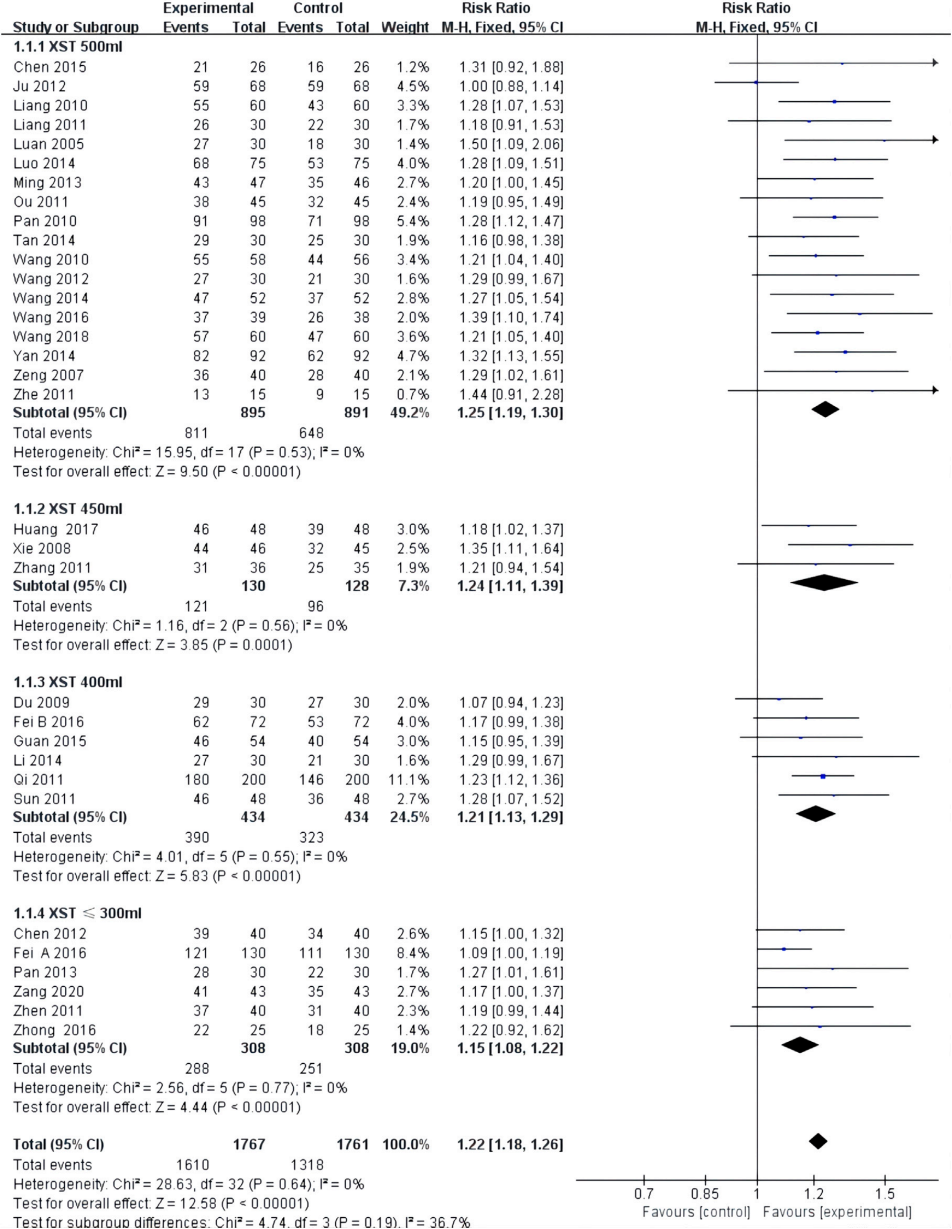
Forest plot of clinical comprehensive efficacy.

Comprehensive clinical efficacy was reported in 33 studies with low heterogeneity among the studies (*I* ^2^ = 0.0%, *p* = 0.64) and was merged with a fixed-effects model. In a subgroup analysis based on XST dosage, the comprehensive clinical efficacy of the combination of CT and 500 mL of XST (RR = 1.25, 95% CI: 1.19–1.30, *p* < 0.00001; Figure 3), 450 ml of XST (RR = 1.24, 95% CI: 1.11–1.39, *p* = 0.0001; Figure 3), 400 ml of XST (RR = 1.21, 95% CI: 1.13–1.29, *p* < 0.00001; Figure 3), and less than 300 ml (RR = 1.15, 95% CI: 1.08–1.22, *p* < 0.00001; Figure 3) was significantly different from the control group.

#### 3.4.2 Secondary efficacy outcomes

##### 3.4.2.1 ECG improvement

**FIGURE 4:**
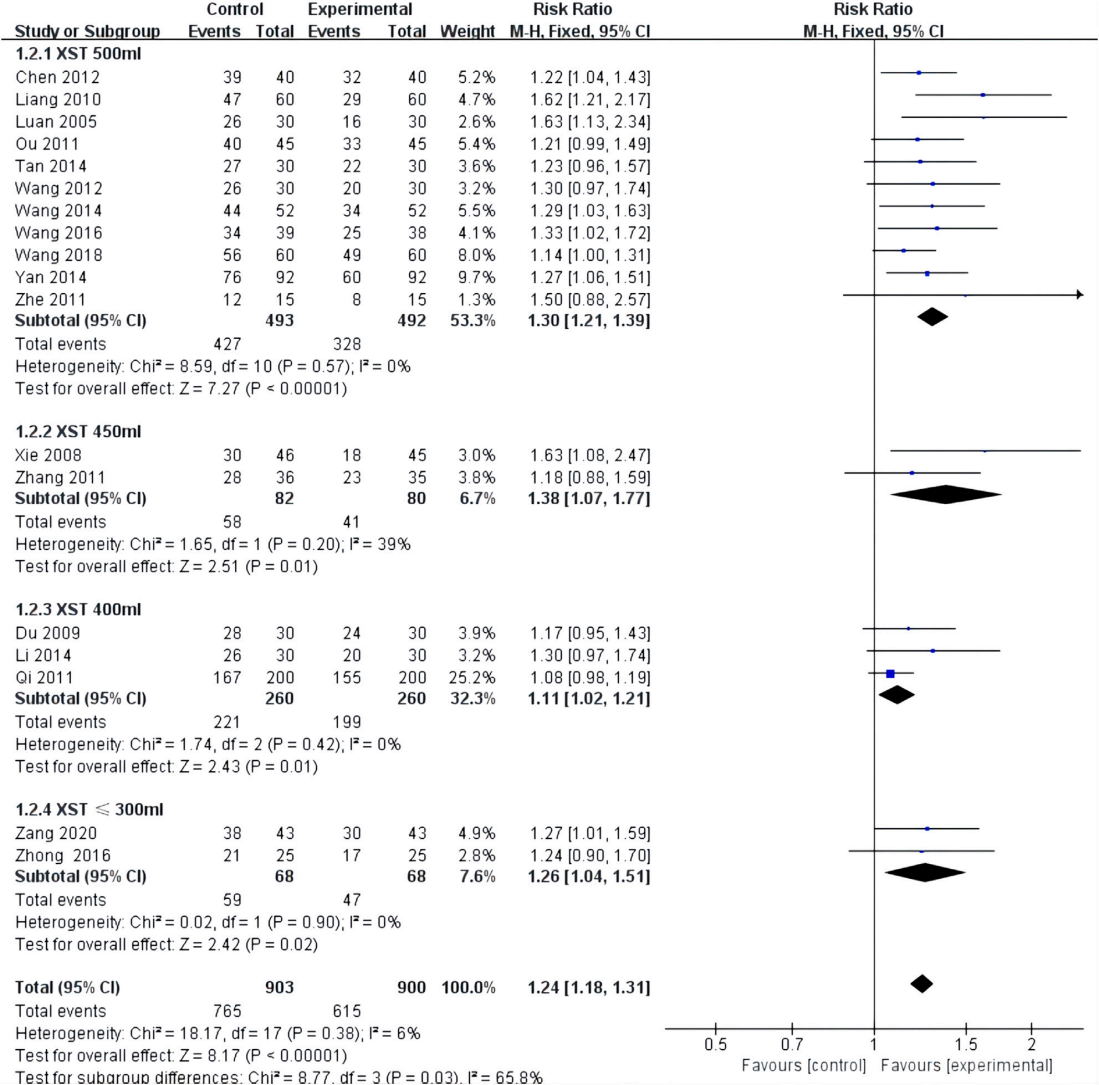
Forest plot of ECG Improvement.

ECG improvement was reported in 18 studies with low heterogeneity among the studies (*I* ^2^ = 0.0%, *p* = 0.54) and was merged with a fixed-effects model. In a subgroup analysis based on the dose of XST, the ECG improvement by the combination of CT with 500 mL of XST (RR = 1.30, 95% CI: 1.21–1.39, *p* < 0.00001; Figure 4), 450 mL of XST (RR = 1.38, 95% CI: 1.07–1.77, *p* = 0.01; Figure 4), 400 ml of XST (RR = 1.11, 95% CI: 1.02–1.21, *p* = 0.01; Figure 4), or less than 300 ml (RR = 1.26, 95% CI: 1.04–1.51, *p* = 0.02; Figure 4) was significantly different from the control group.

##### 3.4.2.2 Frequency of angina attacks

**FIGURE 5:**
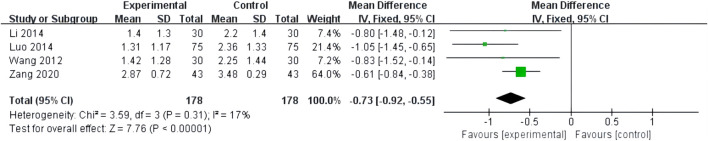
Forest plot of frequency of Duration of angina pectoris.

The frequency of angina attacks was reported in four studies with low heterogeneity (*I* ^2^ = 17%, *p* = 0.31) and was analyzed using a fixed-effects model. The combination of XST and CT decreased the frequency of angina attacks (*MD* = −0.73, 95% *CI:* −0.92 to −0.55, *p* < 0.00001; Figure 5).

##### 3.4.2.3 Duration of angina pectoris

**FIGURE 6:**
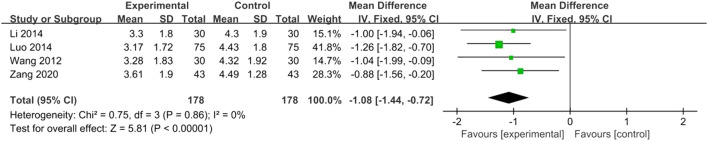
Forest plot of duration of angina pectoris.

The duration of angina was reported in four studies with low heterogeneity (*I* ^2^ = 0%, *p* = 0.86) and was analyzed using a fixed-effects model. The combined use of XST and CT reduced the duration of angina pectoris (*MD* = −1.08, 95% *CI*: −1.44 to −0.72, *p* < 0.00001; Figure 6).

##### 3.4.2.4 Hemorheology levels

**FIGURE 7:**
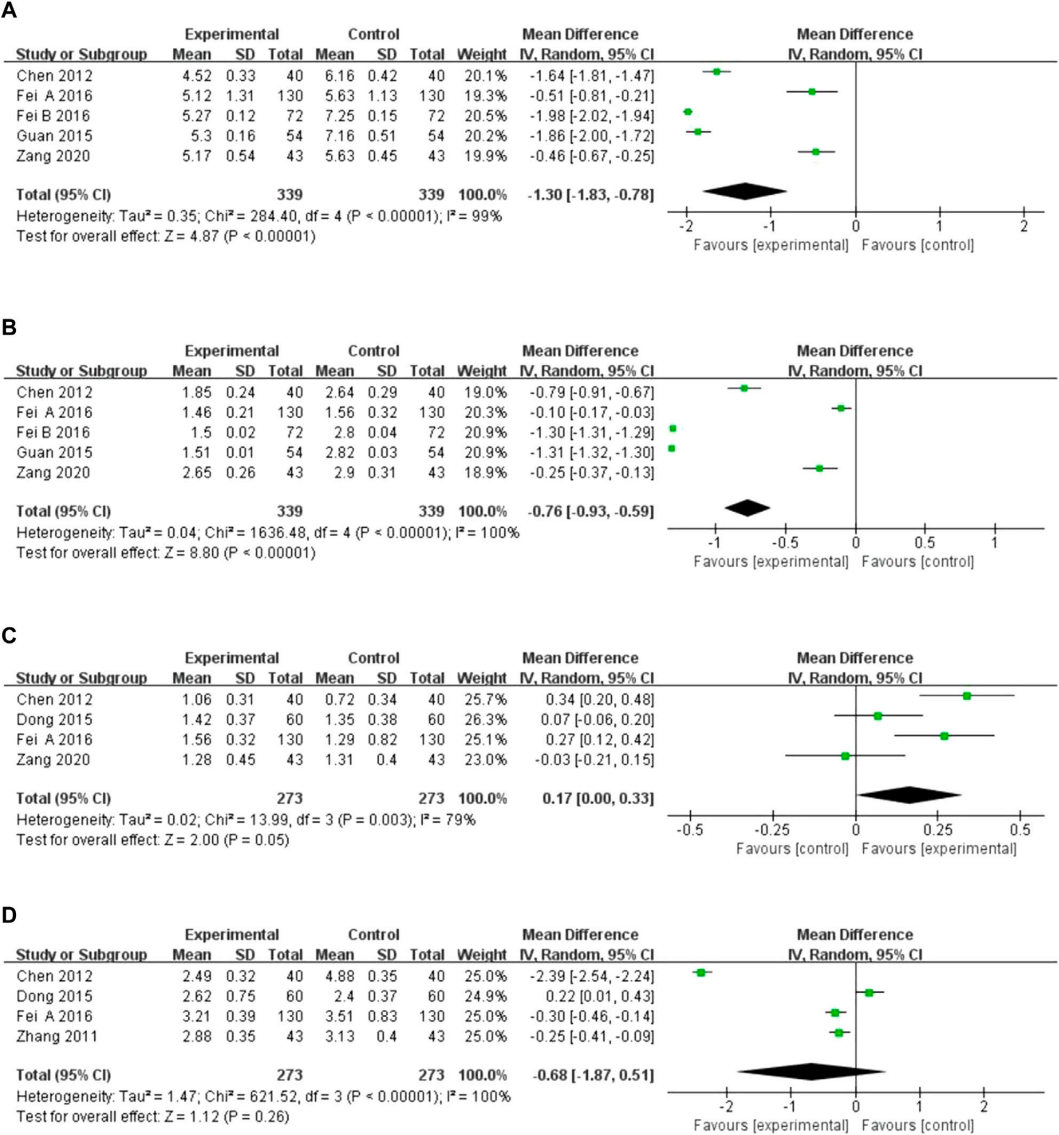
Forest plot of hemorheology leve. **(A)** Total cholesterol. **(B)** Triglyceride levels. **(C)** High-density lipoprotein. **(D)** Low-density lipoprotein.

Five studies reported total cholesterol and triglyceride levels, and four reported high-density lipoprotein (HDL) and low-density lipoprotein (LDL) levels. There was high heterogeneity among the studies (*I* ^2^ = 99%, *p <* 0.00001; *I* ^2^ = 100%, *p <* 0.00001; *I* ^2^ = 79%, *p =* 0.003; *I* ^2^ = 100%, *p <* 0.00001), and the random-effects model was utilized for analysis. The results indicated that XST combined with CT decreased total cholesterol (*MD* = −1.30, 95% *CI:*−1.83 to −0.78, *p* < 0.00001; Figure 7A) and triglyceride levels (*MD* = −0.76, 95% CI: −0.93 to −0.59, *p* < 0.00001; Figure 7B) and increased HDL levels (*MD* = 0.17, 95% *CI* = 0.00 to 0.33, *p* = 0.05; Figure 7C). However, there was no significant difference in LDL levels in patients treated with XST combined with CT compared to CT alone (*MD* = −0.68, 95% *CI*: −1.87 to 0.51, *p* = 0.26; Figure 7D). The greater heterogeneity in the analyses could be due to significant differences in basal lipid levels in the patients included in the studies and differences in XST doses and CT used in the included studies. In addition, the forest plot showed that in terms of total cholesterol and triglycerides, Fei A and Zang studies are more homogenous, and Fei B et al. and Guan et al. studies are more homogenous. This may be because CTs in the studies by Fei A et al. and Zang et al. were very similar, with a similar average age of the patients. In contrast, the CTs used in the studies by Fei B et al. and Guan et al. were similar.

##### 3.4.2.5 Blood lipid levels

**FIGURE 8:**
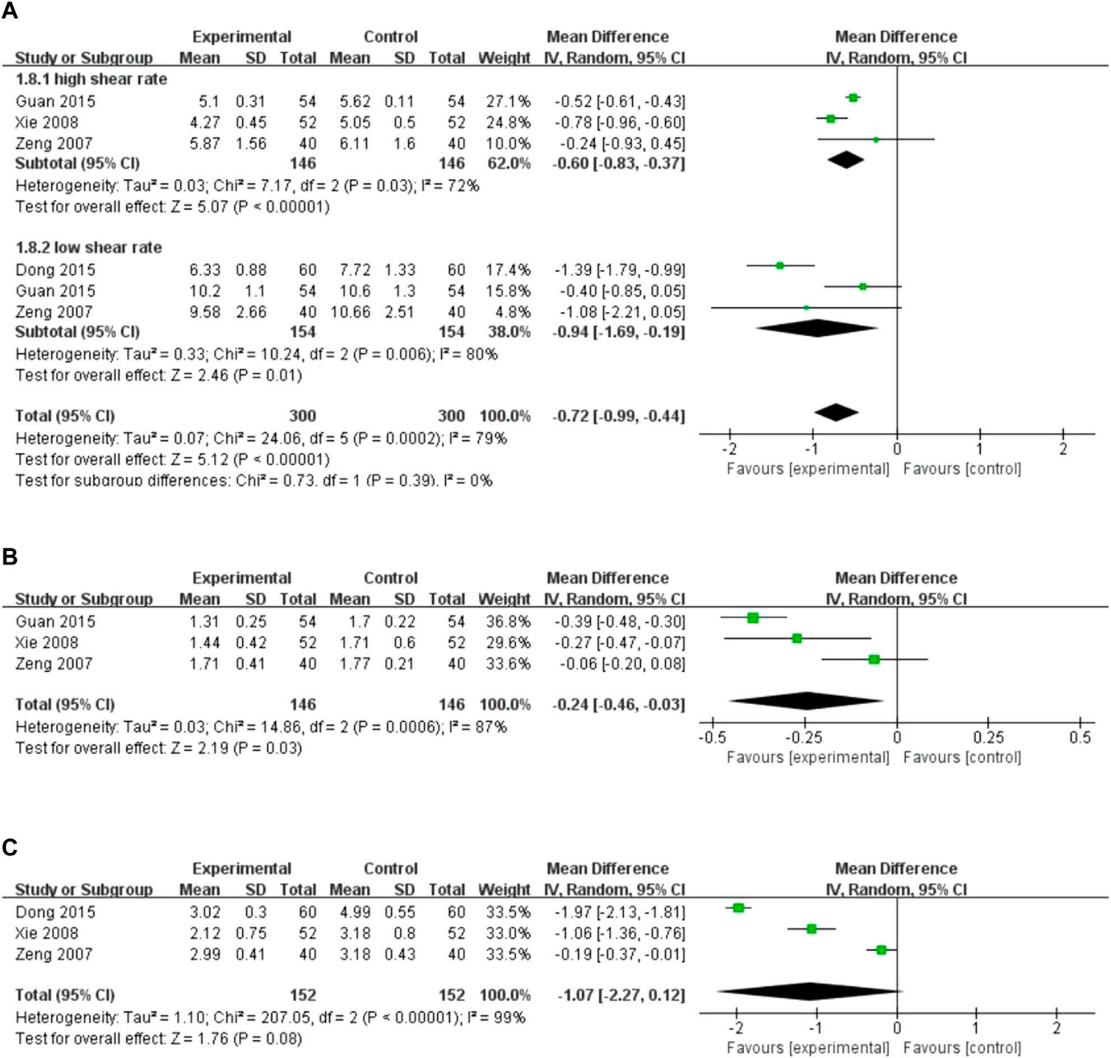
Forest plot of blood lipids level. **(A)** Whole blood viscosity. **(B)** Plasma viscosity. **(C)** Fibrinogen levels.

Four studies reported the level of blood lipid levels assessed by different indicators. There was high heterogeneity among the studies, and the random-effects model was used for the analysis (*I* ^2^ = 72%, *p =* 0.03; *I* ^2^ = 80%, *p =* 0.006; *I* ^2^ = 87%, *p =* 0.0006; *I* ^2^ = 99%, *p <* 0.00001). The results showed that XST combined with CT decreased whole blood viscosity at a high shear rate (*MD* = −0.60, 95% *CI*: −0.83 to −0.37, *p* < 0.00001; Figure 8A), whole blood viscosity at a low shear rate (*MD* = −0.94, 95% *CI*: −1.69 to −0.19, *p* = 0.01; Figure 8A), and plasma viscosity (*MD* = −0.24, 95% *CI*: −0.46 to −0.03, *p* = 0.03; Figure 8B). However, no significant difference in the decreases in fibrinogen levels was observed between the patients who received CT combined with XST and those who received CT alone (*MD* = −1.07, 95% *CI*: −2.27 to 0.12, *p* = 0.08; Figure 8C). Sensitivity analysis was conducted to explore the sources of heterogeneity, but the sources could not be identified. Differences in dosage, duration, and CT regimens among the four included studies could have been responsible for this heterogeneity. Therefore, these results were limited by substantial heterogeneity. More trials with good homogeneity are required to validate the results.

#### 3.4.3 Primary safety outcome

##### 3.4.3.1 Adverse cardiovascular events

**FIGURE 9:**
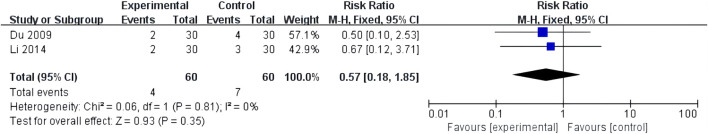
Forest plot of sdverse Cardiovascular Events.

Two studies reported adverse cardiovascular events, with low heterogeneity between the two studies (*I* ^2^ = 0%, *p =* 0.81). The fixed-effects model analyzed the results. The results revealed no significant difference in cardiovascular events between patients treated with XST combined with CT and those treated with CT alone (*RR* = 0.57, 95% *CI* = 0.18–1.85, *p* = 0.35; Figure 9). The details are shown in Supplementary Material S3.

#### 3.4.4 Secondary safety outcomes

##### 3.4.4.1 Adverse events

**FIGURE 10:**
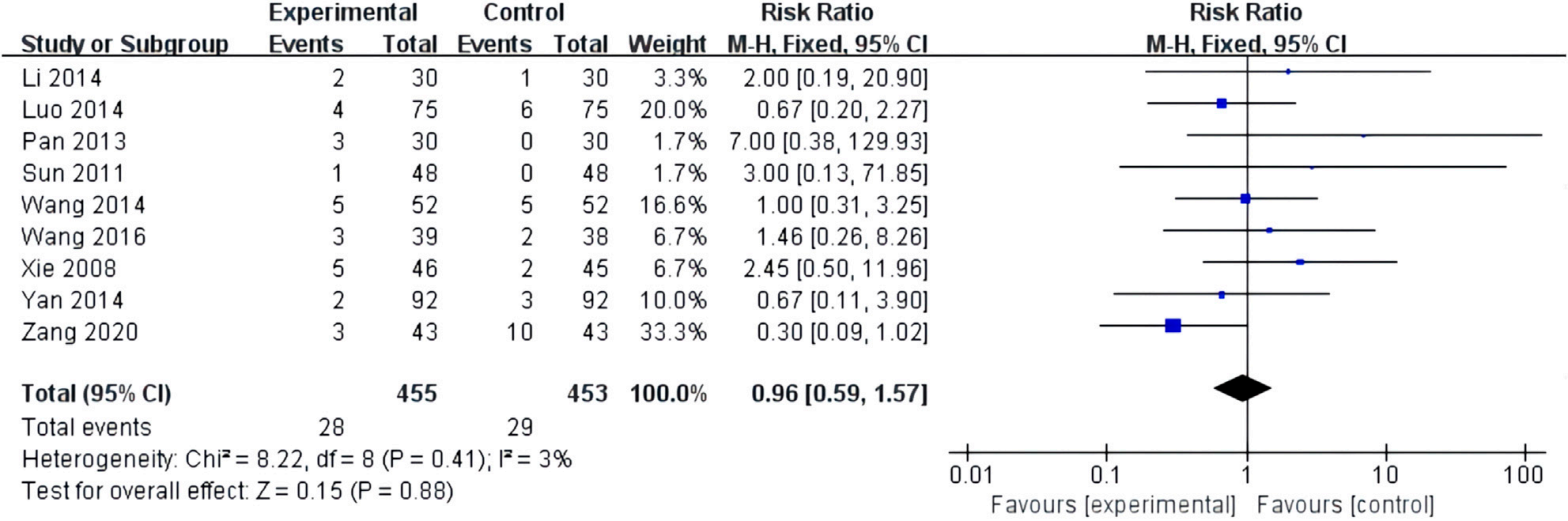
Forest plot of adverse events.

Nine studies reported adverse events with low heterogeneity among the studies (*I* ^2^ = 3%, *p* = 0.41). The random-effects model helped analyze the adverse reactions, with no significant difference between patients treated with the combination of XST and CT and those treated with CT alone (*RR* = 0.96, 95% *CI* = 0.59–1.57, *p* = 0.88; Figure 10). Our results revealed that gastrointestinal discomfort, tetter, allergy, and subcutaneous ecchymosis constitute the most frequently occurring AEs. These adverse reactions disappeared with symptomatic management. No participants discontinued the study drug due to adverse events. The details are shown in Supplementary Material S4.

### 3.5 Publication bias

**FIGURE 11:**
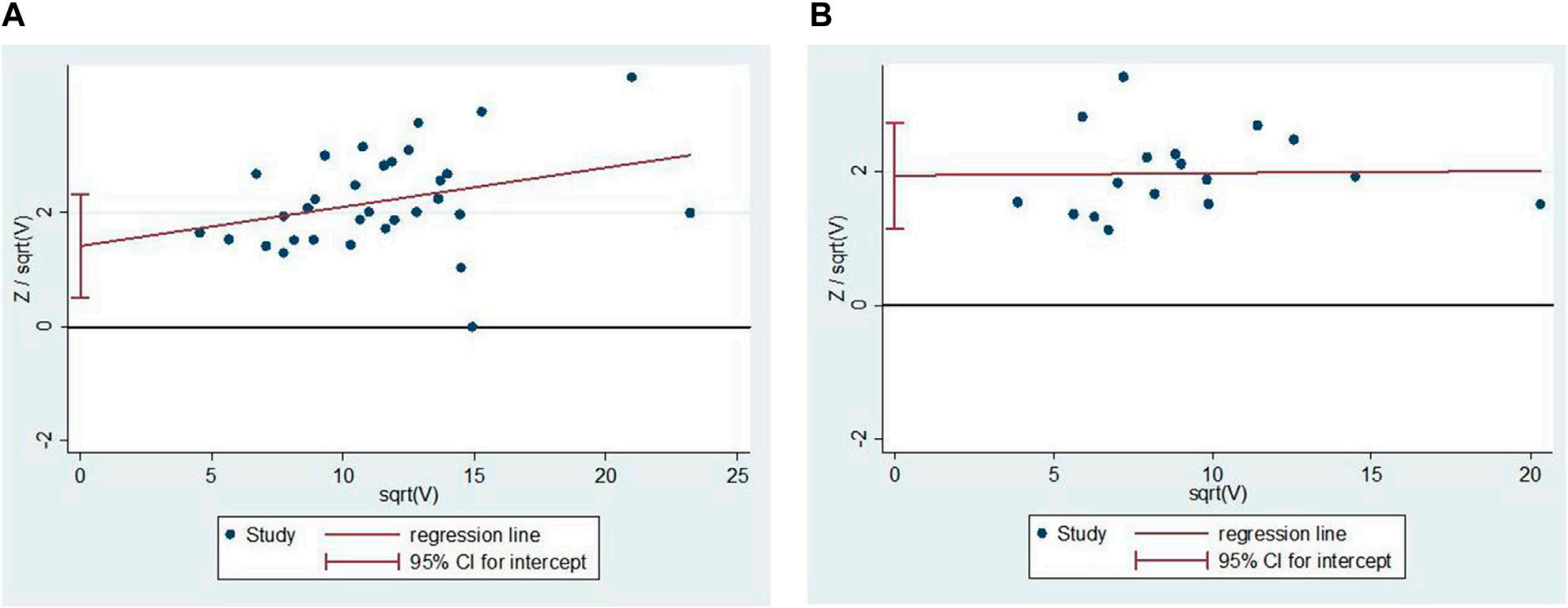
Publication bias. **(A)** The Harbord’s test on comprehensive clinical efficacy. **(B)** The Harbord’s test on ECG improvement.

We analyzed the outcome indicators for more than 10 studies for publication bias. Since the heterogeneity was low, the Harbord test was adopted. The results showed publication bias for comprehensive clinical efficacy (*p* = 0.003; Figure 11A) and ECG improvement (*p* = 0.000; Figure 11B). There were multiple reasons for the biases, including the ease of publication, the retrieval of positive results, the small sample size of the included studies, and a small sample effect. All the included studies in the analysis were in Chinese, so there was language publication bias.

### 3.6 Trial sequential analysis

**FIGURE 12:**
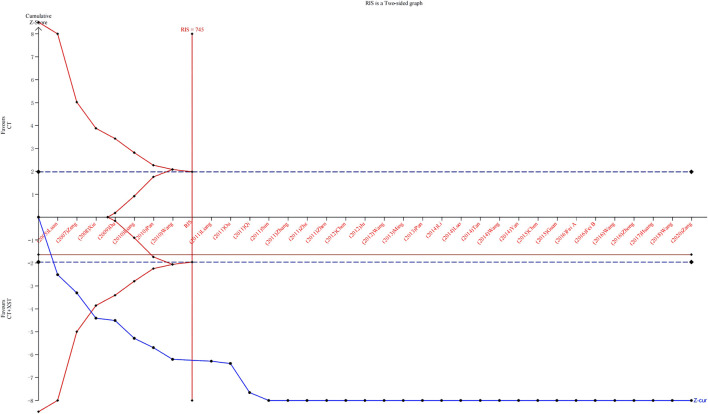
Trial sequential snalysis.

Thirty-three studies reporting comprehensive clinical efficacy were estimated by trial sequential analysis (TSA). Based on the results of related research and this meta-analysis, the class I error definition in this study was set to 0.05, and the statistical efficiency was 0.8. The relative risk reduction (RRR) was 15%, the relative incident rate in the control group was 76.8% (based on the meta-analysis results), and the information axis was set as the cumulative sample size. The TSA analysis showed that the cumulative Z value crossed the traditional boundary value curve (Z = 1.96) and the required information size (RIS) curve. Thus, the cumulative sample size achieved the expected value, the result was stable, and additional tests could not change the conclusion (Figure 12).

### 3.7 GRADE assessment

All outcome indicators were evaluated using the GRADE system. Due to the low risk of bias, accuracy, and heterogeneity, the certainty quality of the evidence was low or very low. An overview of the GRADE evidence is shown in Table 2. The following six reasons cause the serious risks of deviations and reported deviations: 1) No details on the randomization protocol were reported. 2) The implementation of blinding was not reported. 3) There was a lack of allocation concealment. 4) The heterogeneity between studies was high (*p* < 0.1, *I* ^2^ > 50%). 5) Evaluation of the data suggested publication bias, and an equivalent number of “negative” trials may not be included in this study. 6) The sample size included in the study is too small for imprecision. The results of the evidence quality evaluation of the above outcomes provide a reference basis for guiding clinical practice. More largesamples and high-quality RCTs are needed to improve the evidence of the effectiveness of XST in treating UAP.

**TABLE 2:** GRADE evidence profile.

Quality assessment							No of patients		Effect		Quality	Importance
No of studies	Design	Risk of bias	Inconsistency	Indirectness	Imprecision	Other considerations	SXT	Control	Relative (95% CI)	Absolute		
Comprehensive clinical efficacy												
33	Randomised trials	Serious^a^,^b^,^c^	No serious inconsistency	No serious indirectness	No serious imprecision	Reporting bias^d^	1610/1767 (91.1%)	1318/1761 (74.8%)	RR 1.22 (1.18–1.26)	165 more per 1000 (from 135 more to 195 more)	⊕⊕ΟΟ Low	Important
								73%		161 more per 1000 (from 131 more to 190 more)		
Electrocardiogram curative effect												
18	Randomised trials	Serious^a^,^b^,^c^	No serious inconsistency	No serious indirectness	No serious imprecision	Reporting bias^d^	765/903 (84.7%)	620/900 (68.9%)	RR 1.23 (1.17–1.29)	158 more per 1000 (from 117 more to 200 more)	⊕⊕ΟΟ Low	Important
								66.7%		153 more per 1000 (from 113 more to 193 more)		
Frequency of angina attacks (better indicated by lower values)												
4	Randomised trials	Serious^a^,^b^,^c^	No serious inconsistency	No serious indirectness	Serious^e^	None^d^	178	178	—	MD 0.73 lower (0.92–0.55 lower)	⊕⊕ΟΟ Low	Critical
TC (better indicated by lower values)												
5	Randomised trials	Serious^a^,^b^,^c^	Serious^f^	No serious indirectness	Serious^e^	None^d^	339	339	—	MD 1.3 lower (1.83–0.78 lower)	⊕ΟΟΟ Very low	Important
TG (better indicated by lower values)												
5	Randomised trials	Serious^a^,^b^,^c^	Serious^f^	No serious indirectness	Serious^e^	None	339	339	—	MD 0.76 lower (0.93–0.59 lower)	⊕ΟΟΟ Very low	Important
HDL-C (better indicated by lower values)												
4	Randomised trials	Serious^a^,^b^,^c^	Serious^f^	No serious indirectness	Serious^e^	None	273	273	—	MD 0.17 higher (0–0.33 higher)	⊕ΟΟΟ Very low	Important
LDL-C (Better indicated by lower values)												
4	Randomised trials	Serious^a^,^b^,^c^	Serious^f^	No serious indirectness	Serious^e^	None	273	273	—	MD 0.86 lower (0.94–0.78 lower)	⊕ΟΟΟ Very low	Important
Whole blood viscosity—high shear rate (better indicated by lower values)												
3	Randomised trials	Serious^a^,^b^,^c^	Serious^f^	No serious indirectness	Serious^e^	None	146	146	—	MD 0.6 lower (0.83–0.37 lower)	⊕ΟΟΟ Very low	Important
Whole blood viscosity—low shear rate (better indicated by lower values)												
3	Randomised trials	Serious^a^,^b^,^c^	Serious^f^	No serious indirectness	Serious^e^	None	154	154	—	MD 0.94 lower (1.69–0.19 lower)	⊕ΟΟΟ Very low	Important
Plasma viscosity (better indicated by lower values)												
3	Randomised trials	Serious^a^,^b^,^c^	Serious^f^	No serious indirectness	Serious^e^	None	146	146	—	MD 0.24 lower (0.46–0.03 lower)	⊕ΟΟΟ Very low	Important
Fibrinogen (better indicated by lower values)												
3	Randomised trials	Serious^a^,^b^,^c^	Serious^f^	No serious indirectness	Serious^e^	None	152	152	—	MD 1.07 lower (2.27 lower to 0.12 higher)	⊕ΟΟΟ Very low	Important
Duration of angina (better indicated by lower values)												
4	Randomised trials	^a^,^b^,^c^	No serious inconsistency	No serious indirectness	Serious^e^	None	178	178	—	MD 1.08 lower (1.44–0.72 lower)		Critical
Adverse cardiovascular events												
2	Randomised trials	Serious^a^,^b^,^c^	No serious inconsistency	No serious indirectness	Serious^e^	None	6/60 (10%)	12/60 (20%)	—	200 fewer per 1000 (from 200 fewer to 200 fewer)	⊕⊕ΟΟ Low	Critical
								0%		-		
Adverse reactions												
8	Randomised trials	Serious^a^,^b^,^c^	No serious inconsistency	No serious indirectness	No serious imprecision	Strong association	25/425 (5.9%)	27/423 (6.4%)	—	64 fewer per 1000 (from 64 fewer to 64 fewer)		Important
								0%		—		

a No details of the random protocol were reported.

b Didn’t report the implementation of blinding.

c Lack of allocation concealment.

d Evaluation of the data suggested publication bias, and there may be an equivalent number of “negative” trials didn’t be included in this study.

e The sample size included in the study is too small to imprecision.

f The heterogeneity between studies is large (*p* < 0.1, I^2^ > 50%).

## 4 Discussion

Through systematic searching and screening, 34 RCTs were finally included in this meta-analysis to compare the efficacy and safety of XST combined with CT compared to CT alone for treating patients with UAP. We found that the combined use of XST and CT showed improved clinical efficacy in treating UAP by reducing the frequency and duration of angina pectoris. Moreover, it improves blood lipid levels and hemorheology parameter values, with no increases in adverse cardiovascular events or reactions.

A noticeable increase in blood viscosity can cause dyslipidemia, a significant risk factor for coronary atherosclerosis. Atherosclerosis causes coronary artery lumen stenosis or blockage, which leads to temporary myocardial blood supply insufficiency and the production of excess pyruvate, lactic acid, and polypeptides. These can stimulate cardiac autonomic ganglions and eventually cause angina pectoralis symptoms, including precordial pain, chest tightness, and palpitations (Qian et al., 2018; Sincer I et al., 2018; Chen and Wang, 2019). The results of a Chinese cohort study (n = 20,954, age 35–64 years) with a 20-year follow-up revealed (Zhang et al., 2018) that low-density lipoprotein cholesterol (LDL-C) levels (from <1.0 mmol/L to ≥4.1 mmol/L) were significantly and positively associated with the risk of coronary heart disease. The lower LDL-C levels were associated with a lower risk of coronary heart disease over the next 20 years and *vice versa*. Based on the results of this meta-analysis, combined treatment with XST and CT could reduce total cholesterol, triglyceride levels, and whole blood viscosity and increase HDL levels compared to CT alone. Qin et al., (2013) found that XST reduced total cholesterol, triglyceride, and LDL-C levels; increased HDL-C levels, and decreased the degree of aortic intimal lesions in atherosclerotic rabbits. The core drug in XST is *Panax notoginseng*, a typical traditional Chinese medicine used to promote blood circulation and remove blood stasis. Some studies (Han et al., 2017) found that *Panax notoginseng* saponins could inhibit platelet adhesion to the injured endothelial cell surface by inhibiting the expression of vascular cell adhesion molecule-1 (VCAM-1) and the secretion of thromboxane A2 (TXA2) by vascular endothelial cells. *Panax notoginseng* saponins also inhibited the expression of VCAM-1 and the release of thromboxane B2 to inhibit platelet activation and play a role in the prevention and treatment of thrombosis. Another study (Wang, 2020) found that *Panax notoginseng* inhibited thromboxane A2 production by increasing cyclic adenosine monophosphate (cAMP) levels in platelets. Additionally, it inhibited calcium ion and 5-hydroxytryptamine (5-HT) release to inhibit thrombus formation. Acute myocardial ischemia is a direct cause of UAP. Wu et al. (Wu et al., 2015) found that XST could reduce the extent of myocardial infarction, lower serum lactate dehydrogenase, creatine kinase, and creatine kinase isoenzyme levels, promote thrombus lysis, inhibit platelet aggregation, and thus, alleviate myocardial ischemic injury in myocardial ischemia model rats.

The safety indicators for this study were the incidence of adverse cardiovascular events and the incidence of adverse reactions. The incidence of adverse cardiovascular events was studied in 120 patients and experienced by 10% (6/60) of the patients treated with XST plus CT and 20% (12/60) of those treated with CT only. The results showed no statistical difference in the safety of XST combined with CT versus CT alone, indicating that XST combined with CT is safe. The incidence of adverse reactions was studied in 848 patients and was observed in 5.8% (25/425) of the patients treated with XST plus CT. Moreover, the incidence of adverse reactions in patients treated with CT alone was 6.3% (27/423). The main adverse reactions were skin pruritus, rash, subcutaneous ecchymosis, and gastrointestinal reactions. The allergic reactions resolved after taking antihistamines, and the gastrointestinal reactions resolved after slowing the infusion speed. There was no statistical difference in the incidence of adverse reactions between the test and control groups, which indicated that adding XST to CT would not increase the incidence of adverse reactions.

However, this study had some limitations. Most articles did not report the severity of angina pectoris, leading to potential heterogeneity. Several included studies did not report specific randomization methods or allocation blinding. It was also unclear whether randomization or blinding methods were implemented, which may have led to implementation and measurement biases. Blinding can objectively evaluate therapeutic effects, and the quality of the blinding method will directly affect the accuracy of the research results. Therefore, future RCT test designs should refer to the items in the Cochrane Collaboration Network’s risk of bias tool. Moreover, attention should be paid to implementing randomization and blinding methods in designing trials and their execution. The sample size in the included studies was generally small. Only two studies had a sample size of more than 200 cases, and 22 had fewer than 100 cases. The therapeutic index of a study with a smaller sample size may be unstable. The asymmetrical distribution of the funnel chart indicated publication bias, and the subjective biases of the researchers may have exaggerated the therapeutic effect of UAP in the trial group. XST is a drug in *China’s National Catalog of Essential Medicines*, and most of the subjects were Chinese, which may have led to ethnic and geographical biases.

Therefore, the following recommendations were made: 1) The relevant RCT should classify UAP into specific types, including initial angina pectoris, worsening exertional angina pectoris, resting angina pectoris, etc., to ensure targeted conclusions. Simultaneously, the classification of angina pectoris and its course should also be fully and comprehensively reported based on the CONSORT statement (David Moher et al., 2011). This can help identify the source of heterogeneity among studies, carry out subgroup analysis, and analyze the best timing and application of XST. 2) The specific medication category, usage, and dosage of CT must be reported in the primary studies for repeating the procedure. 3) Long-term follow-up after the trials are also necessary, especially for end-point events and safety outcomes. 4) Comprehensive clinical efficacy and ECG Improvement have been reported across most of the original literature. These two indicators are widely used, but the evaluation is difficult to quantify. However, there are few reports on quantifiable indicators, such as the Frequency of Angina Attacks and Duration of Angina Pectoris. Therefore, future research should focus on measurable indicators.

## 5 Conclusion

The available literature data and systematic evaluation methods demonstrated that XST combined with CT improved the therapeutic effect of UPA, relieved angina symptoms, and improved hemorheology parameter values compared to CT alone. No serious adverse effects were observed in this study, and the addition of XST to CT is conditionally recommended. However, a more rigorous RCT should be designed and completed before the widespread use of XST combined with CT is recommended.

## Data Availability

The original contributions presented in the study are included in the article/Supplementary Material, further inquiries can be directed to the corresponding author.

## Abbreviations

ACS, Acute Coronary Syndrome; AE, Adverse Event; cAMP, cyclic Adenosine Monophosphate; CBM, Chinese Biological Medicine Database; CI, Confidence Intervals; CNKI, China National Knowledge Infrastructure; CT, Conventional Treatment; CPM, Chinese Patent Medicine; ECG, Electrocardiograph; HDL, High-density Lipoprotein; ICAM, Intercellular Adhesion Molecule; LDL, Low-density Lipoprotein; MD, Mean Difference; RCT, Randomized Controlled Trials; RR, Relative Risk; RRR, Relative Risk Reduction; RIS, Required Information Size; SR, Sarcoplasmic Reticulum; TSA, Trial Sequential Analysis; TXA2, Thromboxane A2; UAP, Unstable Angina Pectoris; VCAM-1, Vascular Cell Adhesion Molecule-1; VIP, VIP Chinese Sci-tech Periodical Database; XST, Xueshuantong (lyophilized) for injection; 5-HT, 5-hydroxytryptamine.
